# A Review of the Ingredients Contained in Over the Counter (OTC) Cough Syrup Formulations in Kenya. Are They Harmful to Infants?

**DOI:** 10.1371/journal.pone.0142092

**Published:** 2015-11-05

**Authors:** Gabriel Kigen, Naftali Busakhala, Francis Ogaro, Emily Chesire, Nathan Saat, Robert Too, Winstone Nyandiko

**Affiliations:** 1 Department of Pharmacology and Toxicology, Moi University School of Medicine, Eldoret, Kenya; 2 Department of Paediatrics, Moi Teaching and Referral Hospital, Eldoret, Kenya; 3 Department of Pharmacy, Moi University Health Services, Eldoret, Kenya; 4 Department of Epidemiology and Nutrition, Moi University School of Public Health, Eldoret, Kenya; 5 Department of Child Health and Pediatrics, Moi University School of Medicine, Eldoret, Kenya; Nottingham University, UNITED KINGDOM

## Abstract

**Background:**

Cough syrups are widely used in the developing world, but safety of their use in infants and children less than two years has not been well documented. Some syrups contain multiple combinations of such drugs as promethazine, diphenhydramine and ephedrine; which are individually now contraindicated in children less than two years. Despite this, the syrups are available as over the counter drugs and may be dispensed to mothers who are unaware of the potentially hazardous effects to their infants. A descriptive cross-sectional study was used to investigate suitability of cough syrups sold within Eldoret municipality for use in children less than two years of age based on their formulations and available literature.

**Methods:**

Two semi-structured questionnaires were administered to pharmacy attendants and mothers attending sick child clinic at a referral hospital to establish whether cough syrups containing more than one active ingredient of compounds, now contraindicated in children are administered to infants, and awareness of potential serious adverse effects. Data from labeled contents of cough syrups from retail pharmacies was recorded and corroborated with information from literature to determine those deemed to contain the ingredients. The second questionnaire was administered to mothers with children less than two years to ascertain whether they had used the identified syrups. A total of 260 mothers and 55 pharmacy attendants were interviewed.

**Results:**

There was widespread use of the syrups in children, including infants, with 192 (74%) of the respondents having used identified syrups and over 90% of these on children less than 2 years including those less than three months.146 (76%) mothers had administered the syrup at double the recommended dose.

**Conclusion:**

The regulatory authorities should make concerted efforts to discourage use of cough syrups containing ingredients that pose adverse events to infants, including campaigns to educate pharmacy workers and mothers.

## Introduction

Over the counter (OTC) cough and cold preparations are some of the most widely used medications in Kenya, just like in many other parts of the world. They are used in the treatment of common conditions such as allergies including urticaria, allergic rhinitis and common cold in both children and adults [1,2]. They mainly contain combinations of antihistamines, antitussives, decongestants and expectorants, in various proportions. However the benefits of their use in infants has recently generated debate, especially considering that some of the cough preparations containing antihistamines have been associated with serious adverse effects [3–5]. The current view is that antihistamines should not be used in infants, and some researchers have even suggested that 1^st^ generation antihistamines should no longer be dispensed as OTC medicine, owing to their adverse effects [6–8]. In addition, the fact that they are sold as OTC medicine further complicates the issue especially in developing countries whereby regulation of drugs is still of major concern. In fact, the American College of Chest Physicians (ACCP) and US Food and Drug Administration (FDA) have both recommended that OTC cough and cold products should not be used in infants and children under 2 years old [9,10].

There are several generic syrup preparations available in the Kenyan drug shops from various manufacturers which contain more than one active ingredient which are sold as OTC drugs. These include those containing a combination of up to three anti allergic drugs; promethazine, diphenhydramine and ephedrine in various proportions as the main active ingredients. These preparations are quite popular because they are cheap and readily available. However the rationale for the use of a combination of three drugs with similar functions in one product is unclear. In addition, promethazine and ephedrine have serious toxicity profiles. The products do not also contain warning labels barring their use in infants, despite the fact that all the three drugs are contraindicated in children less than two years [11–14]. Some even contain an extra antihistamine, chlorpheniramine to make a total of four antiallergic drugs in one preparation.

Promethazine is a first-generation antihistamine with strong sedative, antiemetic and anticholinergic properties. It is widely used as an antihistamine, a sedative as well as antiemetic [15]. However serious adverse effects including fatal respiratory depression, sleep apnea, oversedation, agitation, seizures, hallucinations, and dystonic reactions have been reported with its use. Other adverse effects include cardiac arrests and neuroleptic malignant syndrome [16]. This led to withdrawal of its use in children less than 2 years in America in 2004, and strengthened warning (boxed warning) in children over 2 years [12,13,17–19]. Promethazine is therefore not recommended to be used in children less than 2 years, and this warning is contained in the manufacturers manual [20].

Diphenhydramine is a first-generation antihistamine with anticholinergic, antitussive, antiemetic and sedative properties that is mainly used to treat allergies and common cold. It is also used in the management of drug-induced Parkinsonism and other extrapyramidal symptoms. The drug has a strong hypnotic effect and has been approved as a non-prescription sleep aid [21].The adverse effects associated with diphenhydramine include sedation, motor incoordination, epigastric distress, dizziness and thickening of bronchial secretions. Case report studies conducted in USA regarding diphenhydramine monotoxicity concluded that the most common symptoms for all cases were cardiac dysrhythmias, seizure activity, and/or sympathetic pupil responses. The most common autopsy finding was pulmonary congestion [11,22,23]. This led to its contraindication in neonates, premature infants and breastfeeding mothers [24].

Ephedrine is a sympathomimetic drug used as a decongestant and bronchodilator in treatment of allergic disorders, asthma and hypotension associated with anaesthesia. It is also an appetite suppressant, concentration aid and a stimulant. Ephedrine is found in various plants in the genus *Ephedra* and is structurally similar to epinephrine, phenylpropanolamine and methamphetamine, an illicit drug of abuse. The drug has therefore been clandestinely used as a performance enhancing drug and to manufacture methamphetamine [25–27]. Its adverse effects, especially associated with an overdose include nervousness, insomnia, vertigo, headache, tachycardia, palpitations and convulsions. Serious adverse effects have been observed with its use in children [14]. Orally administered ephedrine is therefore only recommended for use in children less than 12 years under prescription as per the manufacturer’s instructions [28–30].

From the existing literature, it is therefore inappropriate that three drugs which are individually not recommended, or recommended to be used with caution in children, can be combined in one formulation and administered freely to children, including infants. The aim of our research was to investigate the chemical formulations of the OTC cough preparations in the Kenyan market and to establish the safety of their use in children, especially infants; based on currently available literature. We also determined the rate and frequency of their use in children less than two years and the factors influencing the choice of these drugs. Additionally, we sought to ascertain whether the attendants working in the retail pharmacies are aware of the fact that cough syrups containing some specific ingredients should not be administered to children less than two years.

## Materials and Methods

Permission to conduct the research was obtained from the Institutional Research and Ethics Committee [31], of Moi Teaching and Referral Hospital (MTRH) and Moi University (*approval ref FAN*:*IREC 1211)*.All efforts were made to protect the privacy and anonymity of the participants throughout the study. The committee allowed the use of verbal consent from the mothers/caretakers of the children as the study was only descriptive with minimal risk to the subjects and did not require disclosure of their identities. Across-sectional descriptive study was adopted, with all the mothers/caretakers attending outpatient paediatric clinic with children less than 2 years of age, and pharmacy outlets within Eldoret municipality included in the study. Mothers attending paediatric clinic with children over 2 years were excluded. Any other drug preparations administered to the children that were not cough syrups such as antibiotics, antipyretics and pain killers were also excluded. Simple random sampling was used to determine the target sample of the pharmacies, while systematic sampling procedure was employed in order to identify the number of mothers required for the study. We obtained information from the Ministry of Health that the number of registered pharmacies in Eldoret town then was 127. Fisher’s formula was used to determine the number of pharmacies to be included in the study, and a sample size of 55 was arrived at. Similarly, the equation was used to calculate the desired sample size of the mothers to be included in the study. From the MTRH records, average the number of mothers attending the pediatric clinic is about 2000 per month, with 800 having children less than 2 years. Using the formula then, the desired sample size was calculated to be 260.

### Data collection

This was done through questionnaires and semi structured interviews which were administered to the participants. In order to establish whether the syrups were used by infants and children less than two years, two sets of questionnaires were used to collect data on cough syrup use from both pharmacies and mothers attending the sick child clinic at MTRH (S1 and S2 Figs). The first questionnaire was administered to attendants working in the pharmacies to ascertain the composition of the cough syrups in their premises, and to select those whose formulation fitted into the category of cough syrups containing more than one active ingredient, or contraindicated in infants. It was also used to establish the rate and frequency of use of these syrups; and to ascertain whether the attendants were aware of risks associated with their use in children, especially infants. The composition and strength of the syrups, brand names, cost, batch numbers and country of origin, manufacturer, and labeled contents of all the cough syrups available in the outlets were recorded. The toxicity profiles of the formulations were then corroborated with information available from literature, and each of the syrups identified to fit in the category of cough syrups containing more than one of the now contraindicated active ingredients was then photographed and anonymized.

The second questionnaire was administered to mothers attending the outpatient pediatric clinic at MTRH to ascertain whether they had used any of the identified syrups, or any other cough preparations that were deemed to be contraindicated in infants based on the results from the first questionnaire. The photographs obtained from the first questionnaire, or empty bottles/packets were used assist the mothers in the identification of the syrups. Details of the type of syrup administered, age of child when the syrup was administered, dosage administered and reason for purchase of the syrup were subsequently recorded. The frequency of the syrup use in children less than two years of age was also recorded.

### Statistical analysis

Cough syrups were grouped according to their ingredients. The ingredients and quantities were determined and compared to the recommended levels in order to ascertain their suitability. For each category of syrups, the frequency of use in children aged less than two years was determined. The extent to which cough syrups containing these ingredients was administered to children less than 2 yrs, and frequency of use of these drugs was then established. The level of awareness of the pharmacy staff about the risk predisposed by use of these drugs on children less than 2 years was also determined. A chi-square test was used to assess the association between caretaker’s level of education, type of drug used and dosage levels at 5% level of significance.

## Results

A total of 260 mothers attending the sick child clinic at MTRH, and attendants from 55 pharmacies at Eldoret municipality were interviewed. The compositions, strengths, labeled instructions and costs of the identified syrups are as illustrated in Table 1.

**Table 1:** List of the compositions of identified syrups cough syrups containing more than one active ingredient.

	Cough syrup				Cost (Ksh)		
Syrup	Diphenhydramine HCl	Promethazine HCl	Ephedrine HCl	Chlorpheniramine	60 ml	100 ml	Instructions on the bottle
**A**	5mg	2.5mg	7.5mg		45	60	- Children under 3yrs-2.5ml 2 to 3 times daily- Children 3–12 yrs - 5ml 3times daily
**B**	5mg	2.5mg	7.5mg		60	90	- Children below 2 yrs - Not recommended - Children 2–6 yrs - as prescribed by physician
**C**	5mg	2.5mg	7.5mg		50	90	- Children 1-6yrs - 2.5ml 3–4 times a day- Children 7-12yrs - 5ml 3–4 times a day
**D**	5mg	2.5mg	5mg	2.5mg	60	90	- Children 2-5yrs-2.5ml every 6 to 8 hours - Children 6–12 yrs - 2.5ml every 4 to 6 hours
**E**	5mg	2.5mg	7.5mg		60	90	- Children 2-5yrs - 2.5mls 3–4 times a day or as directed by physician- Children 6–12yrs - one 5mls 3 to 4 times a day
**F**	5mg	2.5mg	7.5mg		50	80	- Children 2-6yrs - 2.5ml every 3–4 hours or as directed by physician- Children 6-12yrs - 5ml 3–4 hours
**G**	5mg			2mg	60	80	- Children - 2.5ml to 5ml every six hours or as recommended by the doctor
**H**			7.5mg	2mg	60	90	- Children under 2yrs -Not recommended - Children 2–6 yrs - As prescribed by the physician
**I**	10mg	5mg			50	70	- Children 2–6 yrs - As directed by the physician- Children 6 to 12 yrs - 5ml 3–4 times a day

The syrups were identified by alphabetical letters (A-G). A total of nine syrups were identified to contain a combination of promethazine, diphenhydramine and chlorpheniramine in various proportions. Out of these, five (A, B, C, E & F) contained 2.5 mg of promethazine, 5 mg of diphenhydramine and 7.5mg of ephedrine per 5mls. Syrup D had a lower amount of ephedrine (5mg) but had in addition 2.5mg of chlorpheniramine to make a total of four active ingredients in one preparation. Syrups G, H &I had two ingredients each; diphenhydramine and chlorpheniramine in G, ephedrine and chlorpheniramine in H and diphenhydramine and promethazine in I. Syrup C had instructions allowing for use in children under two years (2.5ml three to four times daily), syrup A in those under 3 years (2.5ml two to three times daily), whereas all the others had instructions for use in children aged two years and above. The average cost the syrups was about one US dollar (90 Kenya Shillings) per100ml (Table 1). All the identified syrups were manufactured in Kenya, but we also got to learn that most of the ingredients were imported, mainly from India.

192(74%) of the respondents had administered at least one of the identified syrups to their babies, with the most popular being syrup A with 91(36%) having administered (Table 2).

**Table 2:** Details on the sex of the children, highest level of their mother’s education and proportions of those who have used any of the identified syrups, as well as the type of syrup.

Question		Number	%
Sex of the baby	M	141	**54**
	F	119	**46**
Mother’s highest level of education	1°	59	**23**
	2°	128	**49**
	3°	73	**28**
Ever administered any of the identified syrups to baby?	Yes	192	**74**
	No	68	**26**
Listed Syrup	A	91	**36**
	B	36	**15**
	C	27	**11**
	D	23	**9**
	E	14	**5**
	F	9	**4**
	G	9	**4**
	H	5	**2**
	I	5	**2**
	A&D	18	**7**
	A&B	14	**5**

**1°** - Primary school level of education

**2°** - Secondary school level

**3°** - Tertiary level (university, technical colleges etc).

The syrups were mainly administered to infants belonging to the age bracket of 6–12 months (96, 50%), but notably (82, 43%) were administered to those aged 3–5 months, including infants as young as 2 months (5, 2%). Nine (5%) of the children who used the identified syrups were in the age bracket of between one and two years. The most popular dosage was one teaspoonful three times a day, with 146(76%) of the respondents having administered, which is double the recommended dose of2.5ml three to four times daily.

There was no significant association between the dosage use and level of education of mother/care giver (*χ* ^2^ = 7.105, df = 4, p = 0.131). 92% of the mother’s with primary level of education administered a dose of one teaspoonful three times a day whereas 80% of those with secondary education and 50% of those with tertiary education used the same dose. Advice from the pharmacy attendants was identified as the leading factor which influenced the choice of syrup (Table 3).

**Table 3:** Factors that influenced choice of the syrup.

	Reason for purchase	Number	%
A	Advice from the pharmacy attendant	155	**43**
B	Cost	68	**19**
C	Efficacy, based on past use	32	**9**
D	Other reasons (e.g. friend told her)	9	**3**
A&B		55	**15**
A&C		18	**5**
B&C		14	**4**
A,B&C		14	**4**

## Discussion

Cough and cold products have been used in paediatric patients for many years to provide relief for the uncomfortable symptoms. However, several reports from trials conducted in children have concluded that there are no significant benefits of using the syrups over placebo. On the contrary, this may predispose the children to serious adverse effects, especially those less than two years of age [32–36]. To this extent, the regulatory authority in America in January 2008 withdrew some of the OTC cough products, or restricted their use in children, especially those under the age of 2 years due to serious and life threatening adverse effects [9,35]. The position of the American Academy of Pediatrics has since then been that the syrups do not work in children less than 6 years, and that their misuse could cause serious adverse effects. Ithas also recommended further research on the efficacy of all cough and cold medications used in children. The Canadian Paediatric Society also holds the same views, according to a report released in November 2011, and both have provided other options for parents and caregivers for the management of cold symptoms including use of saline water, fluid intake, humidified air (vaporizer), honey and acetaminophen if accompanied by fever [37,38].

The current view based on recently conducted studies in several other centres is that the use of cough syrups should be restricted in children owing to concerns on both efficacy and safety, and that other safer remedies such as honey should instead be used [32,39–43]. Authors from a study conducted recently in Kenya arrived at the same conclusions [43,44]. However, despite international warnings, cough and cold medications including those with potentially harmful ingredients are still used in children, including those aged below 2 years in many parts of the world [45,46].

Our findings indicate that OTC cough syrups containing more than one of these ingredients, especially antihistamines are still widely used in Kenya. It is also worth noting that the regulatory body in Kenya, the Pharmacy and Poisons Board (PPB) in its website maintains that OTC cough and cold medicines are not recommended in children less than two years unless with specific prescription from a doctor [47]. The OTC syrups listed as not recommended for children less than six years of age include antitussives (dextromethorphan and pholcodine), expectorants (guaifenesin and ipecacuanha), nasal decongestants (ephedrine, oxymetazoline, phenylephrine, pseudoephedrine and xylometazoline), and antihistamines [brompheniramine, chlorpheniramine, diphenhydramine, doxylamine, promethazine and tripolidine] [47]. Despite this, our findings indicate that they are not only still in use in children under six years, but also in multiple combinations. Of major concern is the dosage whereby 76% of our respondents had administered the syrups at a dose of 5mls three times a day, which is a high dose even if the syrup was suitable for the infants. Parental misunderstanding including poor numeracy skills has been described as one of the components leading to overdosage and toxicity of OTC cough syrups [48].

Most of the pharmacy attendants whom we interviewed were not comfortable when we inquired about their level of education. This could be attributed to the fact that there was an ongoing crackdown on unlicensed pharmacies at the time by the regulatory authorities. Many (60%) were however not aware that the ingredients we sought were harmful to children, especially when administered in combinations. According to them, the most important factors which influenced the sale of a specific syrup was cost (40%), duration in the market (30%) and the aggressiveness in marketing by the manufacturer (25%). We were informed that some manufacturers provided incentives such as discounts and credit facilities to the outlets. Advertisements of drugs, easy access by patients to community pharmacies and consumer freedom have also been reported as some of the factors contributing to abuse of OTC drugs [49,50].

## Conclusion

The regulatory bodies in Kenya need to revise their list in line with international recommendations and enforce this policy. Production of syrups with multiple antihistamine preparations or other ingredients now contraindicated in infants should also be discouraged. Concerted education or awareness public campaigns on the effects of these syrups should also be undertaken particularly to pharmacy attendants and mothers.

## Supporting Information

S1 Fig Questionnaire 1 (administered to the staff dispensing the drugs at the drug shops). (TIF)

S2 Fig Questionnaire 2 (administered to mothers/caretakers attending MTRH paediatric clinic). (TIF)

## Abbreviations

OTC: Over the counter
MTRH: Moi Teaching and referral Hospital

## References

[1] Knol K (2000) Clinical use of antihistamines in infants and children at risk of asthma development. Allergy 55: 31–34. 10.1034/j.1398-9995.2000.00005.x 10887973

[2] Davila I, del Cuvillo A, Mullol J, Jauregui I, Bartra J, et al (2013) Use of second generation H1 antihistamines in special situations. J Investig Allergol Clin Immunol 23 Suppl 1: 1–16. 24672890

[3] Schaefer MK, Shehab N, Cohen AL, Budnitz DS (2008) Adverse events from cough and cold medications in children. Pediatrics 121: 783–787. 10.1542/peds.2007-3638 18227192

[4] Ryan T, Brewer M, Small L (2008) Over-the-counter cough and cold medication use in young children. Pediatr Nurs 34: 174–180, 184 18543844

[5] Del Cuvillo A, Sastre J, Montoro J, Jauregui I, Ferrer M, et al (2007) Use of antihistamines in pediatrics. J Investig Allergol Clin Immunol 17 Suppl 2: 28–40. 18225708

[6] Church MK, Maurer M, Simons FE, Bindslev-Jensen C, van Cauwenberge P, et al (2010) Risk of first-generation H(1)-antihistamines: a GA(2)LEN position paper. Allergy 65: 459–466. 10.1111/j.1398-9995.2009.02325.x 20146728

[7] Rimsza ME, Newberry S (2008) Unexpected infant deaths associated with use of cough and cold medications. Pediatrics 122: e318–322. 10.1542/peds.2007-3813 18676517

[8] Simons FR (2007) DIphenhydramine in infants. Archives of Pediatrics & Adolescent Medicine 161: 105–105. 10.1001/archpedi.161.1.105-a 17199077

[9] FDA (2008) FDA Releases Recommendations Regarding Use of Over-the-Counter Cough and Cold Products Products should not be used in children under 2 years of age; evaluation continues in older populations http://www.fda.gov/newsevents/newsroom/pressannouncements/2008/ucm116839.htm.

[10] ACCP (2006) American College of Chest Physicians, "Patient Information for Parents of a Child With Cough. http://69.36.35.38/accp/patient-guides/patient-education-guides-cough; " https://www.aacpp.com/pdf/parents/English/Diseases/Cough.pdf ‎Accessed on 17/02/2014

[11] Baker AM, Johnson DG, Levisky JA, Hearn WL, Moore KA, et al (2003) Fatal diphenhydramine intoxication in infants. J Forensic Sci 48: 425–428. 12665005

[12] Buck ML Promethazine: Recommendations for Safe Use in Children: PEDIATRIC PHARMACOTHERAPY. A Monthly Newsletter for Health Care Professionals from the University of Virginia Children’s Hospital. Volume 16 Number 3 3 2010

[13] Starke PR, Weaver J, Chowdhury BA (2005) Boxed warning added to promethazine labeling for pediatric use. N Engl J Med 352: 2653 15972879 10.1056/NEJM200506233522522

[14] Berg A, Furu K, Einen M, Spigset O (2010) [Should children be treated with ephedrine mixture?]. Tidsskr Nor Laegeforen 130: 2474–2475. 10.4045/tidsskr.10.0503 21164588

[15] Braude D, Crandall C (2008) Ondansetron versus Promethazine to Treat Acute Undifferentiated Nausea in the Emergency Department: A Randomized, Double-blind, Noninferiority Trial. Academic Emergency Medicine 15: 209–215. 10.1111/j.1553-2712.2008.00060.x 18304050

[16] Duggal HS, Nizamie SH (2001) Neuroleptic malignant syndrome precipitated by promethazine and lorazepam. Australian and New Zealand Journal of Psychiatry 35: 250–251. 11284912 10.1046/j.1440-1614.2001.0884c.x

[17] Cote CJ, Karl HW, Notterman DA, Weinberg JA, McCloskey C (2000) Adverse sedation events in pediatrics: analysis of medications used for sedation. Pediatrics 106: 633–644. 11015502 10.1542/peds.106.4.633

[18] AAP/COD (1995) Reappraisal of lytic cocktail/demerol, phenergan, and thorazine (DPT) for the sedation of children. American Academy of Pediatrics Committee on Drugs. Pediatrics 95: 598–602. 7700765

[19] FDA (2006) U.S. Food and Drug Administration. Information for Healthcare Professionals—Promethazine (marketed as Phenergan and generic products). FDA ALERT [04/2006]: http://www.fda.gov/Drugs/DrugSafety/PostmarketDrugSafetyInformationforPatientsandProviders/ucm126465.htm. Accessed on 05/07/2013.

[20] PHENERGAN (2010) PHENERGAN—promethazine hydrochloride tablet DESCRIPTION. Wyeth Pharmaceuticals Company. http://dailymed.nlm.nih.gov/dailymed/lookup.cfm?setid=585D516F-9B65-484A-CEBF-EC84D8DD780C. Accesed on 14/06/2013.

[21] Morin CM, Koetter U, Bastien C, Ware JC, Wooten V (2005) Valerian-hops combination and diphenhydramine for treating insomnia: a randomized placebo-controlled clinical trial. Sleep 28: 1465–1471. 16335333 10.1093/sleep/28.11.1465

[22] Nine JS, Rund CR (2006) Fatality from diphenhydramine monointoxication: a case report and review of the infant, pediatric, and adult literature. Am J Forensic Med Pathol 27: 36–41. 16501346 10.1097/01.paf.0000188093.45675.ee

[23] Kuffner E, Patel M (2010) Fatality from diphenhydramine monointoxication: a case report and review of the infant, pediatric, and adult literature. Am J Forensic Med Pathol 31: 106. 10.1097/PAF.0b013e3181c2159c 19918160

[24] BENADRYL (2013) BENADRYL- diphenhydramine hydrochloride injection, solution Parke-Davis Div of Pfizer Inc http://dailymed.nlm.nih.gov/dailymed/lookup.cfm?setid=8878f134-42b9-4c30-b240-2761b6b3aa5f#Contraindications, Accesed on 25/06/2013.

[25] Salocks C, KB K (2003) Office of Environmental Health Hazard Assessment(OHEA): Technical Support Document: Toxicology Clandestine Drug Labs/ Methamphetamine Volume 1, Number 13: EPHEDRINE and PSEUDOEPHEDRINE http://oehha.ca.gov/public_info/pdf/TSD%20Ephedrine%20Meth%20Labs%2010%278%2703.pdf. Accessed on 25/06/2013.

[26] Maglione M, Miotto K, Iguchi M, Jungvig L, Morton SC, et al (2005) Psychiatric effects of ephedra use: an analysis of Food and Drug Administration reports of adverse events. Am J Psychiatry 162: 189–191. 15625222 10.1176/appi.ajp.162.1.189

[27] Avois L, Robinson N, Saudan C, Baume N, Mangin P, et al (2006) Central nervous system stimulants and sport practice. Br J Sports Med 40 Suppl 1: i16–20. 16799095 10.1136/bjsm.2006.027557 PMC2657493

[28] BRONKAID Bayer Corporation. Bronkaid (ephedrine sulfate 25 mg and guaifenesin 400 mg) caplets product information. From BayerCare website. http://www.bronkaid.com/en/faqs/index.php. Accesed on 26/06/2103.

[29] BRONCHODILATE (2012) BRONCHO DILATE (ephedrine hcl guaifenesin) tablet [Contract Pharmacal Corp]. NIH product information http://dailymed.nlm.nih.gov/dailymed/lookup.cfm?setid=3476ce0f-9706-4f7e-a1fd-7d7a83eaa1d8 Accessed on 26/06/2013.

[30] BRONCHOLATE (2007) Broncholate syrup product information (Ephedrine 6.25 mg, Guaifenesin 100 mg per ml) http://products.sanofi.us/broncholate/broncholate.pdf. Accessed on 26/06/2013.

[31] IREC (2014) The Institutional Research and Ethics Committee (IREC) of Moi University College of Health Sciences (MUCHS) & Moi Teaching & Referral Hospital (MTRH) http://irec.or.ke/irecs-role/Accessed on 20/10/2014.

[32] Ashkin E, Mounsey A (2013) A spoonful of honey helps a coughing child sleep. The Journal of Family Practice 62: 145–147. 23520585 PMC3601686

[33] Vernacchio L, Kelly JP, Kaufman DW, Mitchell AA (2008) Cough and cold medication use by US children, 1999–2006: results from the slone survey. Pediatrics 122: e323–329. 10.1542/peds.2008-0498 18676518

[34] Sharfstein JM, North M, Serwint JR (2007) Over the counter but no longer under the radar—pediatric cough and cold medications. N Engl J Med 357: 2321–2324. 18057333 10.1056/NEJMp0707400

[35] Ostroff C, Lee CE, McMeekin J (2011) Unapproved prescription cough, cold, and allergy drug products: recent US Food and Drug Administration regulatory action on unapproved cough, cold, and allergy medications. Chest 140: 295–300. 10.1378/chest.11-0981 21813527

[36] Shefrin AE, Goldman RD (2009) Use of over-the-counter cough and cold medications in children. Canadian Family Physician 55: 1081–1083. 19910592 PMC2776795

[37] AAP (2008) American Academy of Pediatrics. Withdrawal of cold medicines: addressing parent concerns. Available at: http://www.aap.org/en-us/professional-resources/practice-support/Pages/Withdrawal-of-Cold-Medicines-Addressing-Parent-Concerns.aspx. Accessed on September 24, 2015.

[38] Goldman RD, Canadian Paediatric Society, Drug Therapy and Hazardous Substances Committee (CPC) (2011) Canadian Paediatric Society. Treating cough and cold: Guidance for caregivers of children and youth. Paediatr Child Health 2011;16(9):564–6 Posted: Nov 1 2011. Available at http://www.cps.ca/documents/position/treating-cough-cold. Accessed on 24/09/2015. 10.1093/pch/16.9.564 PMC3223897 23115499

[39] Isbister GK, Prior F, Kilham HA (2012) Restricting cough and cold medicines in children. J Paediatr Child Health 48: 91–98. 10.1111/j.1440-1754.2010.01780.x 20598066

[40] Smith SM, Schroeder K, Fahey T (2012) Over-the-counter (OTC) medications for acute cough in children and adults in ambulatory settings. Cochrane Database Syst Rev 8: CD001831 10.1002/14651858.CD001831.pub4 22895922

[41] Vassilev ZP, Kabadi S, Villa R (2010) Safety and efficacy of over-the-counter cough and cold medicines for use in children. Expert Opin Drug Saf 9: 233–242. 10.1517/14740330903496410 20001764

[42] Chang CC, Cheng AC, Chang AB (2014) Over-the-counter (OTC) medications to reduce cough as an adjunct to antibiotics for acute pneumonia in children and adults. Cochrane Database Syst Rev 3: Cd006088 10.1002/14651858.CD006088.pub4 24615334 PMC11023600

[43] Waris A, Macharia WM, Njeru EK, F E (2014) Randomised Double Blind Study to Compare Effectiveness of Honey, Salbutamol and Placebo in Treatment of Cough in Children with Common Cold. East African Medical Journal Vol 91,. 26859020

[44] Standardmedia (2014) Why honey, not cough syrup, is best remedy for your child, http://www.standardmedia.co.ke/evewoman/article/2000139696/why-honey-not-cough-syrup-is-best-remedy-for-your-child. Accessed on 27/10/2014. STANDARD Digital, Standard Group 2014

[45] Sen EF, Verhamme KM, Felisi M, t Jong GW, Giaquinto C, et al (2011) Effects of safety warnings on prescription rates of cough and cold medicines in children below 2 years of age. Br J Clin Pharmacol 71: 943–950. 10.1111/j.1365-2125.2010.03860.x 21564162 PMC3099382

[46] Lazarus SG, Lanski SL, Smith AS, Simon HK (2013) Cold preparation use in young children after FDA warnings: do concerns still exist? Clin Pediatr (Phila) 52: 534–539. 23539689 10.1177/0009922813482761

[47] PPB (2014) Children’s over-the-counter cough and cold medicines: advice to parents; Department of Pharmacovigilance, Pharmacy and Poisons Board(Kenya), http://pharmacyboardkenya.org/?page_id=399, Accessed on 27/10/2014.

[48] Lokker N, Sanders L, Perrin EM, Kumar D, Finkle J, et al (2009) Parental misinterpretations of over-the-counter pediatric cough and cold medication labels. Pediatrics 123: 1464–1471. 10.1542/peds.2008-0854 19482755 PMC2911576

[49] Cooper R (2013) Surveillance and uncertainty: community pharmacy responses to over the counter medicine abuse. Health Soc Care Community 21: 254–262. 10.1111/hsc.12012 23320510

[50] Cooper RJ (2013) Over-the-counter medicine abuse-a review of the literature. Journal of substance use 18: 82–107. 23525509 10.3109/14659891.2011.615002 PMC3603170

